# Data Processing Approaches to Measure Velocity of Electromagnetic Gun on Laser Screen in Complex Environment

**DOI:** 10.3390/s22176573

**Published:** 2022-08-31

**Authors:** Huiyan Hao, Wenyu Liu, Peng Xu, Hui Zhao

**Affiliations:** School of Information and Communication Engineering, North University of China, Taiyuan 030051, China

**Keywords:** velocity measurement, laser screen, Ensemble Empirical Mode Decomposition, Correlation Algorithm

## Abstract

The exit velocity of the armature is an important indicator in measuring the launching performance of the electromagnetic gun. The non-contact photoelectric detection technology with the use of a laser screen was applied to the measurement of the armature velocity of the electromagnetic gun. By means of taking the signals that pass through the laser screen obtained by the velocity measurement system as the research object, we solved problems such as the harsh test environment of the launch armature velocity of the electromagnetic gun, the interferences on the armature signal passing through the laser screen unavoidably caused by various factors such as vibration, electromagnetic interference, shock wave, flare, smoke and fragments, and even the non-recognition of the signal passing through the laser screen in severe cases. A data-processing algorithm that combines the Ensemble Empirical Mode Decomposition (EEMD) with Correlation Algorithm (CA) was proposed, with the aim of processing the signals passing through the laser screen, while using the maximum slope point as the time passing through the laser screen so as to calculate the velocity of the armature passing the laser screen. This method can effectively reduce the influence of interference on the test results, and the test results from two sets of velocity measuring systems show that the velocity obtained by the proposed approach is highly consistent.

## 1. Introduction

The electromagnetic gun is a new type of weapon developed with electromagnetic rail launch technology [1]. The launching velocity of the armature determines the initial kinetic energy imparted by the electromagnetic gun. The velocity measurement is of great significance for measuring the efficiency of the electromagnetic launching system, the R&D and type approval of the electromagnetic gun, and corresponding theoretical research on ballistics [2]. Therefore, the measurement of the movement velocity of the armature of the electromagnetic gun is the most important test component among its various movement parameter tests.

Non-contact photoelectric detection technology with the use of a laser screen is applied to the measurement of the velocity of the high speed objects, due to its unique advantages such as high test accuracy, fast response velocity, and wide application range [3]. Since the high launch velocity of the armature of the electromagnetic gun may produce fragments during the launch process, meanwhile accompanied by interferences such as the strong flare, vibration, smoke, and magnetic field changes, the test environment is very complicated. During the feature extraction of the signal of the armature passing through the laser screen, the test data will be disturbed by various factors and even the effective signal passing through the laser screen sometimes is overwhelmed by the interference. In order to eliminate the interference to obtain the effective signal of the armature passing through the laser screen, and to accurately extract the time information of the armature passing through the laser screen, it is necessary to find the optimal algorithm to filter out the interference and improve the accuracy in selection of the feature points of the test data, thereby improving the accuracy of the test system. A data processing method for measuring the velocity of the electromagnetic gun on the laser screen, especially in the complicated environment that combines the Ensemble Empirical Mode Decomposition (EEMD) with Correlation Algorithm (CA), is proposed in this paper on the basis of analyzing the experimental data and extracting the signal characteristics.

## 2. Signal Acquisition and Processing of Laser Screen Armature Velocity Measurement

**Figure 1:**
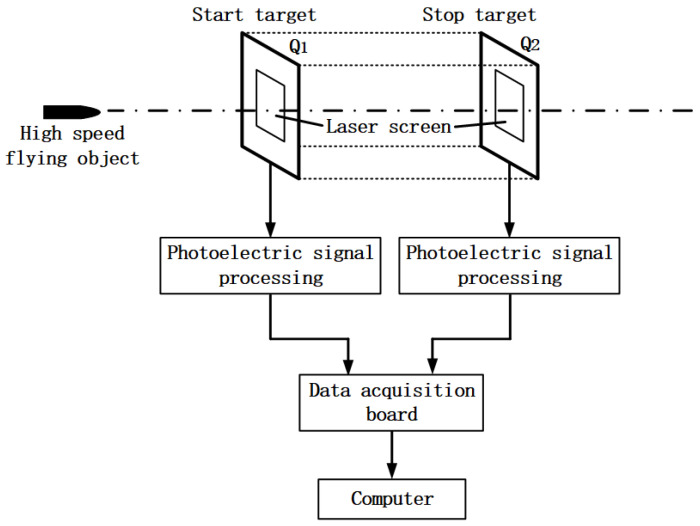
Schematic diagram of the laser velocity measurement system.

By using the principle of zone intercept velocity measurement, we quantified the armature velocity. The speed parameters of a high speed flying object are measured by a laser screen. The time interval of the pulse signal is generated when the high speed flying object that passes through two laser screens is recorded. Then the velocity of high speed flying object passing through the screen is calculated from the calibrated target distance, which is the distance between two laser screens. The laser screen was composed of a semiconductor laser, retroreflector, and photodetectors. After undergoing collimation and one-dimensional beam expansion, the laser beam emitted by the laser transmitting module irradiates the retroreflector and is reflected by the retroreflector. The convergence is carried out by the laser receiving module so that it forms the laser screen [2]. Ultimately the laser beam is received by the high-speed photoelectric detection module. The speed measurement system has two laser screens with a certain parallel distance to each other, which consists of the starting target and the stopping target, so as to realize the effective measurement of the high speed flying object passing the targets [2]. The test system for the signal of armature utilizes two semiconductor laser units as the light source to form two parallel laser screens with an identified distance through the optical system. It consists of a zone-block device including a start target and a stop target [2]. When the armature passes through the two laser screens respectively, the photoelectric detection module converts the light of the blocked part and the variable quantity of the luminous flux into electrical signals. The two-channel signals passing through the laser screen are collected by the data acquisition card and then sent to the computer. When processed by the dedicated software for velocity measuring, the time interval (τ) of the armature passing through the two laser screens can be obtained. From the accurately measured distance (*s*) between laser screen $Q_{1}$ and $Q_{2}$, v=s/τ, the velocity of the armature passing through the midpoint of the laser screen area can be obtained. The test principle of the signal of armature passing through the laser screen is shown in Figure 1.

**Figure 2:**
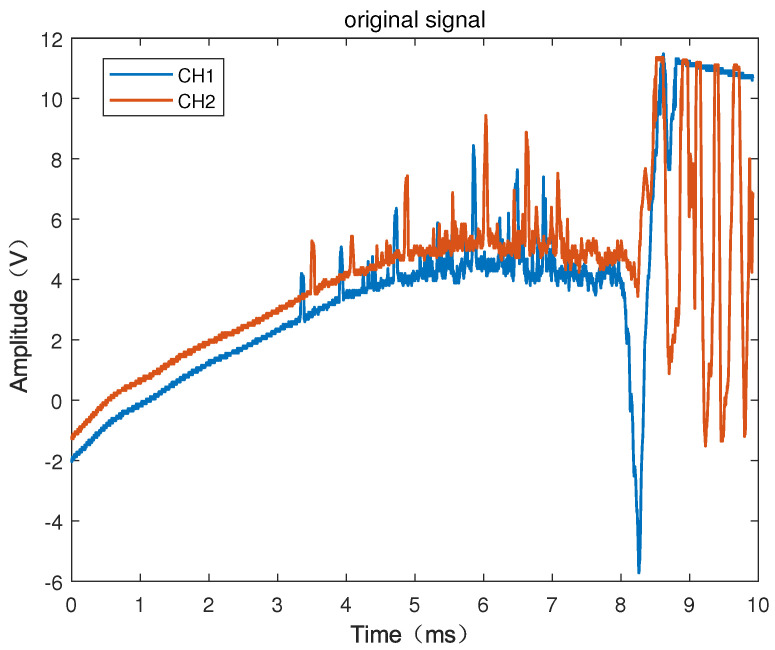
The two-channel signal of the six-series armature passing through the laser screen.

**Figure 3:**
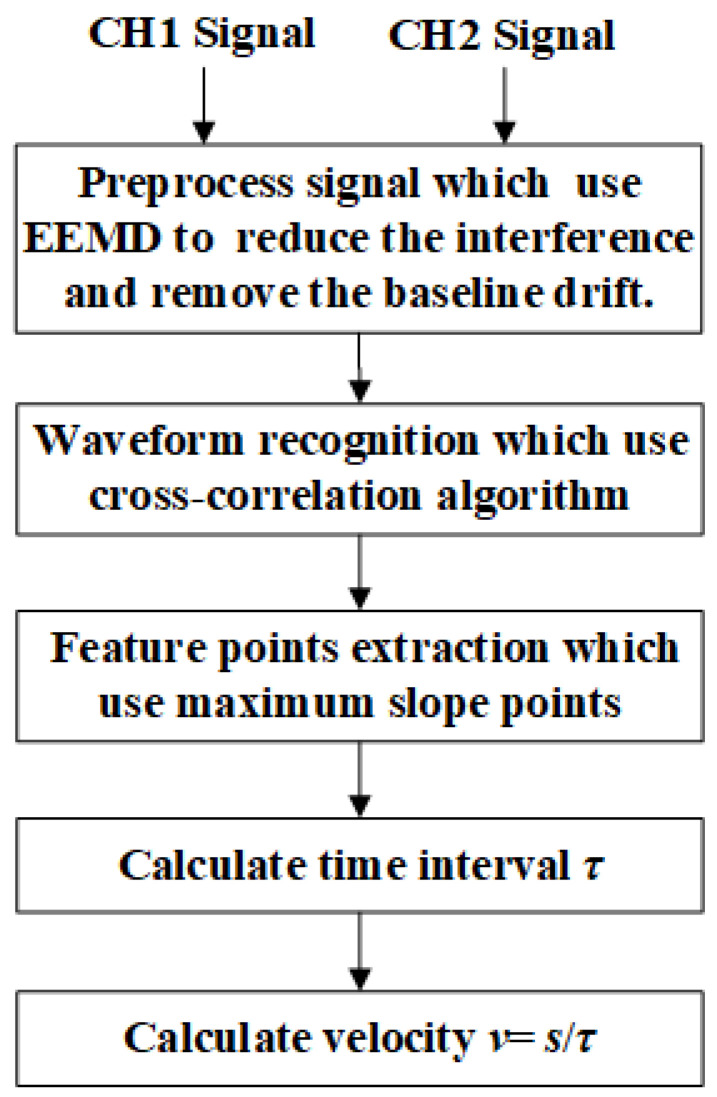
Data processing flow.

Figure 2 is the waveforms of the six-series armatures passing through the two laser screens obtained by the high-velocity acquisition card. It can be seen from the figure that the signals contain the vibration interference caused by the launch of the electromagnetic gun to the vibration of the test system. The negative pulse with a very wide amplitude indicates that it has been interfered with by a stronger flare and then the signal returns, so the pass target pulse is superimposed on the wider negative pulse or positive pulse, resulting in the baseline drift of the signal that passes through the laser screen. The signal peaks from the two channels do not appear in pairs due to many fragments or debris generated during the armature launching process, when the armature is counter weighted with a nylon bracket within the experiment, especially when there are several peaks with small amplitudes. It is difficult to judge whether a peak is particle interference, debris, or small fragments only by observation. When firing the electromagnetic gun, the ground will vibrate greatly under the action of recoil, causing the vibration of the laser screen to generate large interference. The amplitude of the interference caused by vibration was estimated to be more than two times that of the effective pulse. Generally, it is slower than that of the armature passing through the laser screen, so the propagation velocity of the vibration often appears after the signal passes through the laser screen, so that it is far away from the muzzle. A greater vibration attenuation would cause less interference. The signal generated by the vibration of the laser screen can be determined as an invalid signal by using the correlation calculation due to the great difference in shape and amplitude of signal armature. From the complexity of the interference subject to the armature, we know that the passing-through signal of the armature is a typical non-stationary random signal with very complex signal components. For such signals, the traditional time-domain localization analysis is quite difficult to carry out. Therefore, a passing-through signal of armature processing method that combines EEMD and CA is proposed for this non-stationary signal. First, the EEMD is used to preprocess the signal to reduce the interference of high-frequency noise in the passing-through signal and remove the baseline drift. Then, the correlation algorithm is used to analyze and identify the denoised signal to obtain an effective passing-through signal of the armature. Finally, the point with the maximum slope in the passing-through signal is selected as the feature point to obtain the moment that the armature passes through the laser screen. According to the ratio of the distance *s* between the two laser screens and the time interval τ of the armature passing through the two laser screens, the flying velocity *v* of the armature is obtained. The specific steps of the data processing method that combines EEMD and CA are shown in Figure 3.

## 3. Signal Preprocessing of Armature Passing through the Laser Screen

The regular denoising methods for the signal of armature passing through the laser screen generally include wavelet transform, empirical mode decomposition techniques, and so on. The wavelet technology has the characteristics of multi-resolution analysis and good time-frequency localization. Nevertheless, it is still a linear transformation in essence. The appropriate wavelet basis can be selected according to the characteristics of the signal, but the results obtained by the analysis on the different wavelet basis adopted to the same signal are very different. Thus, it is still a difficult point to choose the wavelet basis according to the criteria balancing the current theory and practical application [4,5]. Empirical Mode Decomposition (EMD) is a fully data-driven adaptive decomposition method which can decompose the signal from high frequency to low frequency into the sum of a finite number of physically meaningful intrinsic mode functions (IMFs) and the remainder. In essence, EMD method starts from the characteristic time scale [6,7]. First, we separate the modes with the smallest characteristic time scale, then we separate the modal functions with the larger characteristic time scale. Finally, the components with the largest characteristic time scale are separated. Therefore, the EMD method can be treated as a set of high-pass filters or a sieving process. The sieving of EMD not only eliminates the superposition of modal waveforms, subsequently decomposing the signal into multiple IMF components, it but also smooths out uneven amplitudes and avoids large amplitude differences between adjacent waves. Therefore, the EMD can be treated as a set of adaptive high-pass filters, and the bandwidth and cutoff frequency of the filters change with the signal. IMF components can be amplitude modulated or frequency modulated. Variable instantaneous amplitude and frequency greatly improve the efficiency of signal decomposition, making EMD very suitable for the analysis and processing of non-stationary nonlinear signals. Despite the above merits, it retains problems in terms of modal aliasing, under-envelope, end-point effect and iteration number, which need to be further improved in order to better reveal the internal real characteristics of the signal [6].

Ensemble Empirical Mode Decomposition (EEMD) is an adaptive processing approach for non-stationary signals [8,9], which can solve the disadvantages, in which the accuracy of the wavelet algorithm depend on the selection of the wavelet basis. Moreover, it adds the white noise assistance based on the EMD method, permitting to solve the modal aliasing problem that may be caused by EMD decomposition [10].

### 3.1. EEMD Algorithm

**Figure 4:**
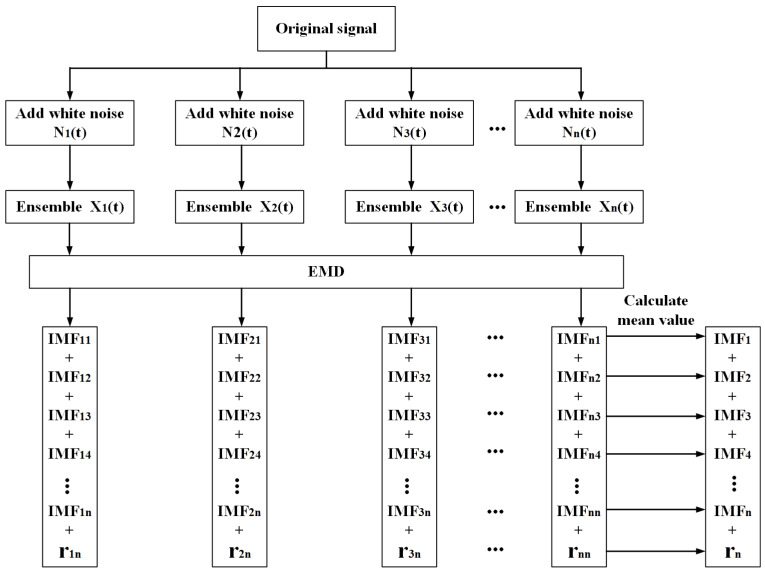
The process of EEMD algorithm.

The decomposition principle of EEMD is as follows: when the added white noise is uniformly distributed over the whole time-frequency space, this time-frequency space is composed of different scale components divided by the filter bank. For each EMD decomposition, the added white noise is uniformly distributed over the entire time-frequency space, while the different frequency scales of the signal are automatically projected onto the corresponding frequency scales in the uniform time-frequency space established by the white noise. Since different white noises are added for each EMD decomposition, the noises are uncorrelated, and the artificially-added noise is offset when evaluating the overall average of all the corresponding IMFs by EMD decompositions. As a whole, the mean value will eventually be considered the true result, the only durably fixed part is the signal itself, and the multiple tests being added are aimed at removing the redundant noise [10,11,12,13,14]. The process of the EEMD algorithm is shown in Figure 4.

The steps of the EEMD-based algorithm to remove high-frequency noise and baseline drift are as follows:

Step1: Add a group of white noise w(t) to the original signal x(t) to form an ensemble X(t).

(1) X(t)=x(t)+w(t)

Step2: Carry out EMD decomposition for ensemble X(t) to obtain the each order IMF, EMD decomposition steps are shown in Algorithm of EMD.

(2) $$X\left(t\right)=\sum_{i=1}^{n}c_{i}\left(t\right)+r\left(t\right)$$

Step 3: A group of different white noises $w_{j}\left(t\right)$ were added to the analyzed signal x(t), in order to obtain another ensemble $X_{j}\left(t\right)$. After EMD decomposition of $X_{j}\left(t\right)$, each order of IMF was obtained.

(3) $$X_{j}\left(t\right)=\sum_{i=1}^{n}c_{ji}\left(t\right)+r_{j}\left(t\right)$$

Step 4: Calculate the mean of each IMF and take it as the final IMF.

(4) $$c_{j}\left(t\right)=\frac{1}{N}\sum_{i=1}^{n}c_{ji}\left(t\right)$$

where *N* represents the number of adding white noise, i.e., the number of the ensembles, which obeys the statistical law of Formula (5), where ϵ is the amplitude of noise, and $\epsilon_{n}$ is the error of the original signal and the signal obtained by summation of the final IMF.

(5) $$\epsilon_{n}=\frac{\epsilon }{\sqrt{N}}$$

When the noise amplitude ϵ is constant, the more often white noise is added, the more fidelity in the final result will be reached, and the better completeness of EEMD will be achieved [10,13,14].

Step 5: Remove high-frequency interference and zero drift that is the low-frequency trend term; each IMF component is calculated by the previous step. IMF1 and IMF2 have a high frequency which occupies little energy and is evenly distributed in the whole signal. Most of them are high-frequency noises introduced in measurement. The lowest frequency IMF component $IMF_{n}$ and trend term $r_{n}$ are regarded as low frequency interference (including baseline drift interference). The pre-processed signal can be obtained by adding up the remaining IMFs, such as the Formula (6). The signal has a large amplitude and contains most of the energy of the over-target signal. The signal reconstruction can remove the interference of high frequency vibration and zero drift. Certainly, according to the signal characteristics, IMF components that we are in interested in can be selected and superimposed as the reconstructed pre-processing signals.

(6) $$Y\left(t\right)=IMF3+\cdots +IMF_{n-1}$$

Detailed procedures of EMD are summarized as follows:

Step1: Firstly, all extremum points of decomposed signals x(t) are determined and then the cubic spline difference function is used to fit them, to obtain the average values $m_{1}\left(t\right)$ of upper and lower envelope lines.

Step2: Subtract the mean from the signal, in order to obtain an oscillatory mode $h_{1}\left(t\right)$ as Formula (7).

(7) $$h_{1}\left(t\right)=x\left(t\right)-m_{1}\left(t\right)$$

Step3: If $h_{1}\left(t\right)$ obeys the stoppage criteria, $c_{1}\left(t\right)=h_{1}\left(t\right)$ obtains the first IMF. If not, take $h_{1}\left(t\right)$ as the original signal and repeat Step1 and Step2 to obtain $h_{11}\left(t\right)$ as Formula (8).

(8) $$h_{11}\left(t\right)=h_{1}\left(t\right)-m_{11}\left(t\right)$$

After k times of repeated screening, $h_{1k}\left(t\right)$ meets the stopping criteria, $c_{1}\left(t\right)=h_{1k}\left(t\right)$, $c_{1}\left(t\right)$ contains the highest frequency of the signal. That is the first order IMF.

In order to ensure that the screened IMF has physical significance, Huang et al. proposed the parcauchy convergence criterion, which was realized by limiting Standard Deviation (SD) as Formula (9) [15].

(9) $$SD=\sum_{t=0}^{T}\frac{e{\left(t\right)}^{2}}{r{\left(t\right)}^{2}}=\frac{\sum_{t=0}^{T}{\left|h_{1k-1}-h_{1k}\right|}^{2}}{\sum_{t=0}^{T}{\left|h_{1k-1}\right|}^{2}}<\epsilon$$

*T* is the time length, The value of ϵ is generally between 0.2 and 0.3 [15].

Step4: Remove the first order IMF component $c_{1}\left(t\right)$ of the original signal x(t), the margin $r_{1}\left(t\right)$ is obtained, as shown in Formula (10).

(10) $$r_{1}\left(t\right)=x\left(t\right)-c_{1}\left(t\right)$$

Repeat Steps 1, 2, and 3 to extract signals $r_{1}\left(t\right)$ repeatedly to obtain other order IMFs as Formula (11).

(11) $$\begin{array}{cc} r_{2}\left(t\right)= & r_{1}\left(t\right)-c_{2}\left(t\right)\\ r_{3}\left(t\right)= & r_{2}\left(t\right)-c_{3}\left(t\right)\\ \vdots & \\ r_{n}\left(t\right)= & r_{n-1}\left(t\right)-c_{n}\left(t\right) \end{array}$$

The decomposition result of the original signal x(t) is shown in Formula (12).

(12) $$x\left(t\right)=\sum_{i=1}^{n}c_{i}\left(t\right)+r_{n}\left(t\right)$$

$r_{n}\left(t\right)$ is the residual quantity, which can no longer be decomposed into IMF components and represents the final trend.

### 3.2. Preprocessing of the Signal of Armature Passing through the Laser Screen Based on EEMD

**Figure 5:**
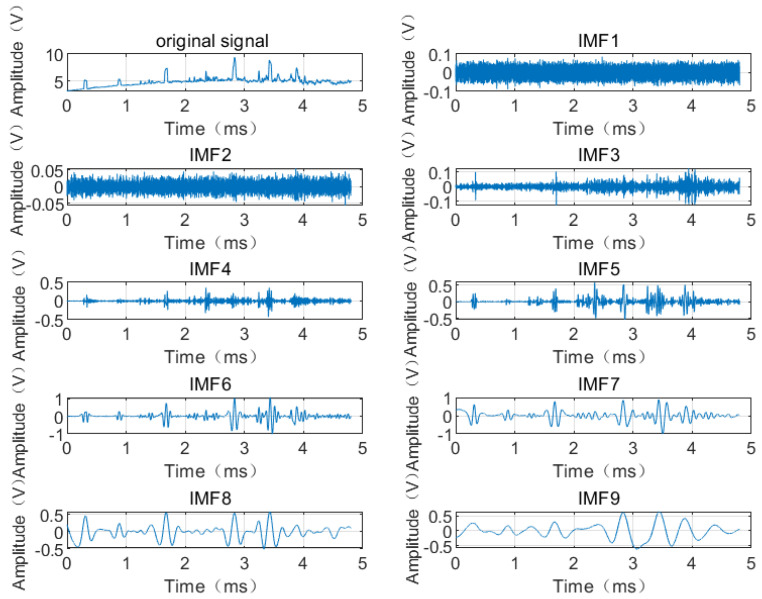
EEMD decomposition result of the signal of the armature passing through the laser screen. Here N=100, the ratio of the standard deviation to the added white noise is 0.1.

The EEMD method is used to decompose the signal of armature passing through the laser screen, so as to obtain a total of nine-order IMF components and residuals, as shown in Figure 5.

**Figure 6:**
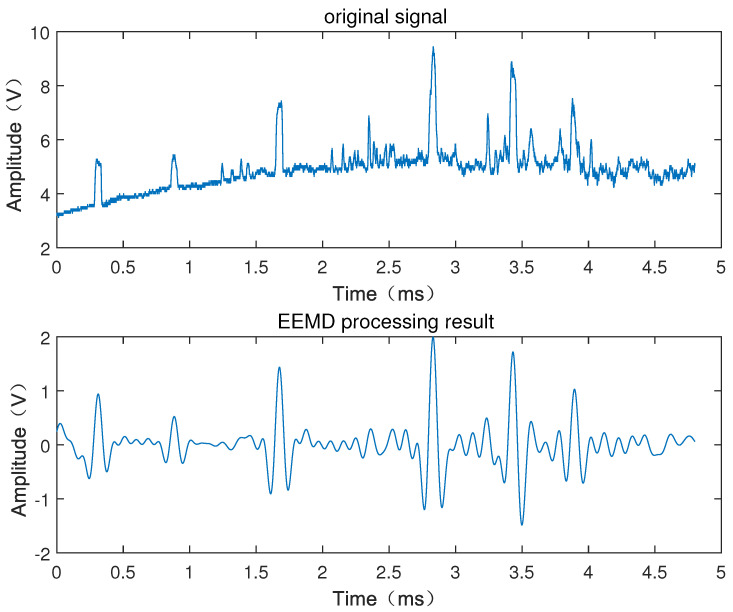
EEMD processing result of the signal of the armature passing through the laser screen.

Figure 5 shows the outcomes derived from the first to the ninth IMF components obtained by adaptive decomposition of EEMD, most of which are in a descending sequence from high frequency to low frequency and have clear physical meanings. IMF1 component has a higher frequency, occupying little energy, is evenly distributed in the entire signal, and most of them are the high-frequency noise being introduced into the measurement; IMF9 is a trend term (i.e., the baseline drift interference); from IMF2 to IMF6, the frequency gradually decreases, but from which it can be clearly seen that when the noise amplitude ϵ is constant, the more times the white noise is added, the more accurate the extracted interference components are obtained, and the more fidelity are with the final result. IMF5~IMF7 are featured by lower frequency and larger amplitude, and contain most of the energy of the signal passing through the laser screen. Therefore, IMF5, IMF6, and IMF7 are selected to reconstruct the signal, so that the signal can be obtained after the noise reduction and baseline removal of the signal passing through the laser screen, as shown in Figure 6:

Figure 6 shows the processing results of the signal of the armature passing through the laser screen, from which it can be seen that the signal of the armature passing through the laser screen after denoising by the EEMD method maintains the original basic shape of the signal, meanwhile reducing the high-frequency interference and the interference of baseline drift in the signal of the armature passing through the laser screen. This process makes the signal of the armature passing through the laser screen clearer.

## 4. Signal Feature Extraction of Armature Passing through the Laser Screen

When denoised by EEMD, a large part of the interference from high-frequency vibration is removed. However, it can be seen from Figure 6 that the interference arising from fragments or particles during the test process has not been removed, which will greatly affect the identification of effective signals. Therefore, the correlation analysis algorithm is proposed in this paper to obtain the corresponding waveforms of the armature in the two channels, which is also the key to the processing of the armature velocity data.

### 4.1. Detection Method of Correlation Algorithm

When the same object passes through two identical laser screens, the signals of this object passing through the laser screen have a strong correlation, as long as the distance between the laser screens is not large.

For two signals x(t) and y(t) with limited signal energy, their correlation functions are defined, as the distance between the laser screens is not large [16].

(13) $$R_{xy}\left(\tau \right)=\int_{-x}^{x}x\left(t\right)y\left(t+\tau \right)dt=\int_{-x}^{x}y\left(t\right)x\left(t+\tau \right)dt$$

of which τ is the time interval of the two signals, and it is obvious that the cross-correlation function of the two signals is a function of the time interval τ.

The estimated value of the cross-correlation function in finite time is as follows.

(14) $$\begin{array}{c} R_{xy}\left(\tau \right)=\frac{1}{\Delta T}\int_{0}^{\Delta r}x\left(t\right)y\left(t-\tau \right)dt=\frac{1}{\Delta T}\int_{0}^{\Delta T}y\left(t\right)x\left(t-\tau \right)dt \end{array}$$

where ΔT is the measured time interval (that is, the width of the signal pulse of the object passing through the laser screen), and the correlation function $R_{xy}\left(\tau \right)$ denotes the similarity of the two signals x(t) and y(t) [14].

In the course of the actual test process, the passing-through waveforms of various armatures collected by the acquisition card are all real finite-length sequences. Hence, a suitable interval can be selected by linear correlation to calculate the correlation coefficient of the two channels, and the size of the correlation coefficient can be used to measure the similarity of the passing-through waveform. This step allows for effectively selecting the feature points, calculating the armature velocity, and improving the system stability. In the test system of the laser screen, the cross-correlation function analysis is carried out for the same armature on the passing-through waveform collected at the start screen and the stop screen, where the corresponding waveform at the maximal value of the correlation coefficient. $R_{xy}\left(\tau \right)$ is the waveform of the stop screen, and the corresponding value is the time interval for the armature passing through the two laser screens, so that the corresponding identification and velocity calculation of multiple armatures can be realized. As such, when the correlation coefficient is greater than 0.9, it is considered that they have achieved correspondence. Therefore, the respective waveforms belonging to each armature can be calculated using this correlation algorithm.

The steps of signal recognition method for the same target passing through two laser screens are as follows:

Step 1: Read the data of the armature pass the laser screen signal, and set a threshold value according to the signal amplitude. The threshold setting is related to the armature section size and other parameters.

Step 2: When the amplitude of the signal is greater than the threshold value, select an appropriate range as the interception length, and select the length according to the speed range and the thickness of the laser curtain. For example, if the armature speed is between 1000 m/s and 2000 m/s, and the thickness of the curtain is 1mm, select the waveform length from from 1 × 10${}^{-6}$ to 2 × 10${}^{-6}$ s.

Step 3: Calculate and compare the correlation coefficients of two over-target signals with the same length, when the value of correlation coefficient is greater than 0.9, it is considered that the waveform of two target signals belong to the same armature or fragment.

The correlation algorithm is based on the similarity degree of signal waveform to determine the correlation coefficient, so it is very effective to automatically calculate the signal attribution of an armature or fragment passing through two laser screens.

**Figure 7:**
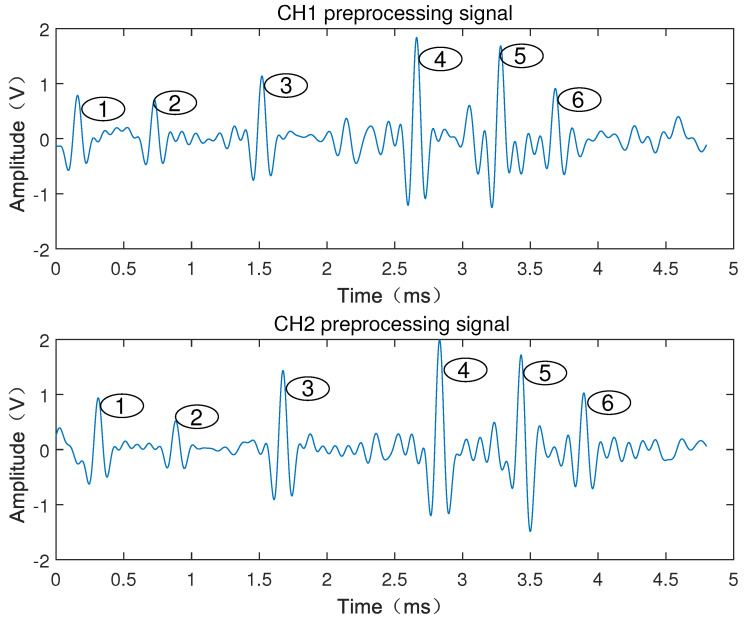
Correlation calculation and analysis results of two-channel signals of six-series armature passing through the laser screen.

Figure 6 shows the passing-through signal when denoised by EEMD, from which it can be seen that the wave peaks with larger or smaller pulse widths do not appear in pairs, where the waveforms with correlation coefficients reaching the threshold can be considered as the passing-through signal of the same object. Figure 7 shows the analysis results of the correlation algorithm, where, if the correlation coefficient does not reach the threshold, it can be judged that they do not belong to the same object, which may be the interference peaks caused by debris or particles. However, as shown in Figure 7, it can be clearly seen that the pulse width of the debris is much narrower than that of the effective signal. The pulse width of the signal is related to the length of the object passing through the laser screen. The length of the debris is much smaller than that of the armature, so the pulse width of the debris is narrower than that of the effective signal passing through the laser screen. The pulse width threshold can be set to extract the effective signal. It can be seen from Figure 7 that the mark numbers 1–6 are the six pairs of effective passing-through waveforms of the signals of the six-series armature passing through the laser screen.

### 4.2. Determination of Feature Points and Calculation of Armature Velocity

After the previous analysis, Figure 7 shows the results of the signals processed by EEMD decomposition and correlation analysis of the armature passing through laser screen 1 and laser screen 2. The curve of the armature passing through the two laser screens is obtained, from which it can be seen that the waveform passing through the laser screen has a certain pulse width. Therefore, determination of the time period passing through the laser screen is the key to the calculation.

**Figure 8:**
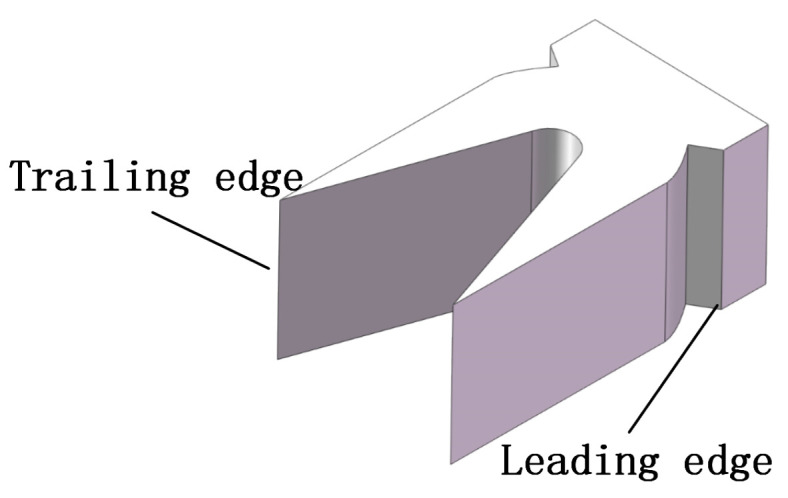
An armature shape.

Due to the particularity of the shape of the armature, the shape of an armature was commonly taken as shown in Figure 8 [17,18]. Its trail section is parallel to the laser screen during the launching process. Therefore, when its trail passes through the laser screen, the waveform slope value reaches the maximum of approximately one. This point can be defined as the feature point of the signal of the armature passing through the laser screen, that is, the maximal slope point is counted as the moment when the armature tail passes through the laser screen, namely, the time interval τ when the armature passing through the two laser screens can be calculated. Finally, the distance *s* between the two laser screens can be measured with the vernier caliper to calculate the velocity *v* of the armature passing through the laser screen each time according tov=s/τ.

The velocity of each armature obtained by using the above-mentioned signal processing, as well as the feature point extraction algorithm to calculate the series launch data, is shown in Table 1.

## 5. Analysis the Reliability of the Method

**Figure 9:**
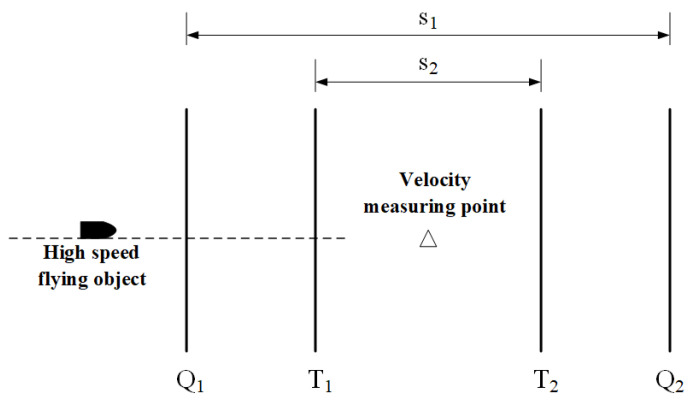
Four laser screens test system.

In order to verify the reliability of the data processing method, two reference laser screens were added during the test, which is placed horizontally along the direction of the ballistic line with the test system, as shown in Figure 9, forming a test system consisting of two sets of zone-block laser screens in velocity measuring systems. On the ballistic axis, with the velocity measuring point as the center, two pairs of laser screen devices $Q_{1}$ and $Q_{2}$, $T_{1}$ and $T_{2}$ are symmetrically set, and the distance between $Q_{1}$ and $Q_{2}$ is set as $s_{1}$ and that between $T_{1}$ and $T_{2}$ is $s_{2}$.

**Figure 10:**
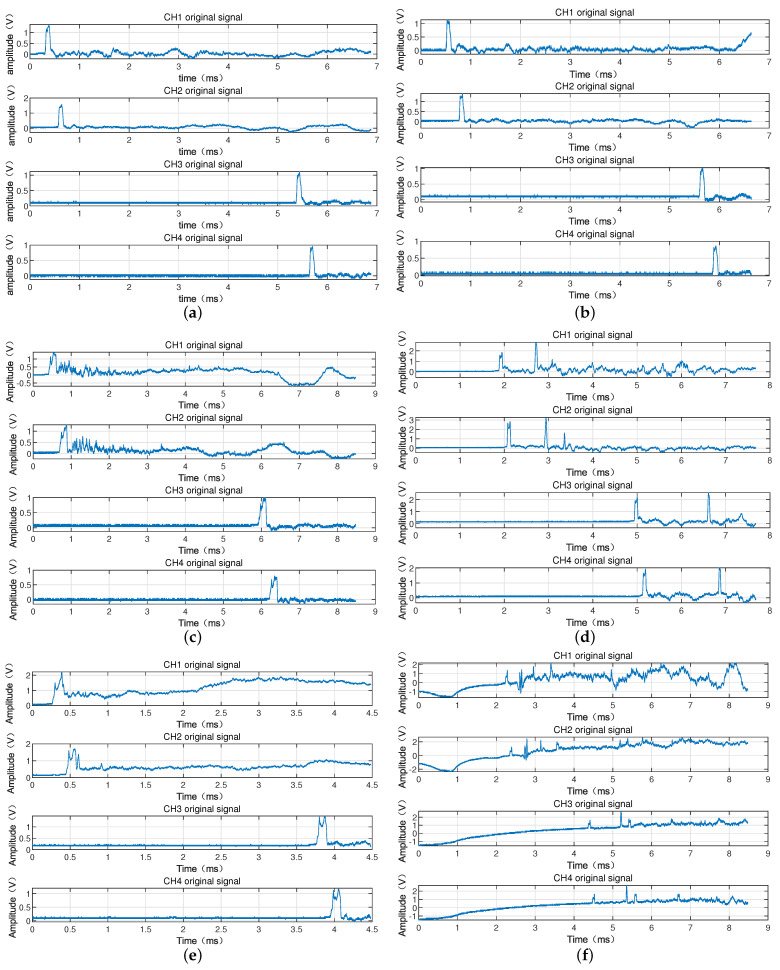
Armature pass through four-laser screen experiment waveforms. (**a**) The first armature passes through four-laser screen waveforms, $s_{1}=4.76$ m, $s_{2}=4.3$ m, L=78.4 mm. (**b**) The second armature passes through four-laser screen waveforms, $s_{1}=4.76$ m, $s_{2}=4.3$ m, L=78.4 mm. (**c**) The third armature passes through four-laser screen waveforms, $s_{1}=4.76$ m, $s_{2}=4.3$ m, L=109.3 mm. (**d**) The fourth armature passes through four-laser screen waveforms, $s_{1}=4.09$ m, $s_{2}=3.63$ m, L=80 mm. (**e**) The sixth armature passes through four-laser screens waveforms, $s_{1}=4.63$ m, $s_{2}=4.17$ m, L=109.3 mm. (**f**) The fourth armature passes through four-laser screen waveforms, $s_{1}=2.315$ m, $s_{2}=2.085$ m, L=109.3 mm.

In order to verify the reliability of the method, we conducted six experiments of an electromagnetic gun launch single armature passing through four laser screens, and obtained the signals of armatures passing through four laser screens. Figure 10 shows the waveforms of electromagnetic gun firing armature passing through four laser screens. Here *L* is the length of the armature.

**Figure 11:**
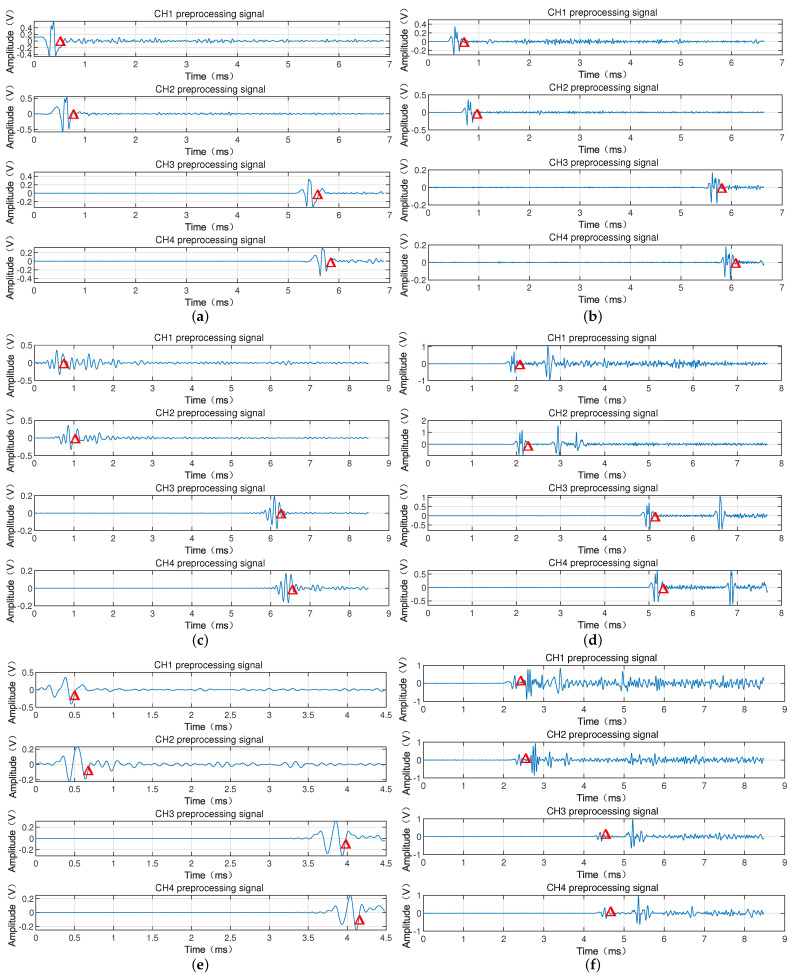
Outcmes from six-times experimental data (**a**–**f**), processed by the proposed algorithm.

It can be seen from Figure 10 that the four channel signals in each experiment include vibration interference generated by the vibration of the test system, as well as interference from small particles and firelight. Figure 10a,b show the waveforms of the electromagnetic gun first shot and the second shot armature passing through the laser screen. The particle and dust in the experimental environment is smaller, and the interference degree of the fire is relatively smaller. Therefore, from the obtained curve, we can obviously obtain the peak of the armature, which was mainly the interference of the laser screens vibration generated from the system vibration and ground vibration. It can be seen from Figure 10b that the amplitude of vibration is larger than that of Figure 10a, and the amplitude of negative pulse is larger, which clearly indicates that the interference from the firelight is more serious. In Figure 10c, in addition to the pulse crest generated by the armature through the laser screen, the signal was also subjected to strong vibration interference, and it also superimposed great vibration on the ground under the action of recoil force during the firing process of the electromagnetic gun, resulting in great interference caused by the vibration of the laser screen. Generally, the propagation speed of vibration is slower than that of armature passing target, so the vibration interference with high amplitude and frequency often appears after the passing target signal. Therefore, it can be seen from the figure that the amplitude of vibration in channels 1 and 2 is larger when they are closer to the muzzle. The larger separation between the muzzle in channels 3 and 4 leads to greater vibration attenuation, subsequently the interference was relatively reduced. In Figure 10d, the four-channel signal pulse waveforms did not appear in pairs. Because the nylon bracket was broken in the experiment, a large fragment amplitude was generated in the armature firing process, even exceeding the amplitude of the light screen through which the armature passed. In Figure 10d, there were several wave peaks with small amplitude, which were the particulate debris generated in the environment after the electromagnetic gun fired several times. There were more small fragments which passed through the laser screens, causing a few small pulse peaks interference. We changed the distance between laser screens and the size of the armature and experimented to obtain the data as shown in Figure 10e,f. It can be seen that the effective passing target pulse is disturbed by vibration, debris or other small particles. Especially, Figure 10f shows the effective pulses was overwhelmed by the interference, the amplitude of the vibration exceeds the effective pulse. The EEMD algorithm is used to remove the interference of noise and baseline drift. Since the signal generated by vibration of the laser screen and waveform generated by other foreign objects passing through the laser screens is different from the armature target signal in shape amplitude, the CA interferences can be treated as invalid signals. Figure 11 shows the processing results obtained through EEMD preprocessing and CA. It can be seen that the signals of all channels in the four groups of experiments were de-noised by EEMD, which reduced a large part of the interference of high-frequency vibration and baseline drift, and the effective signals of armature passing through the laser screens were also extracted by using the CA. In Figure 11, the pulse waveform and the calculation time of the armature passing through the four screens were marked with red triangles.

The algorithm proposed above is used for experimental data processing, and the velocity of the armature passing through the midpoint of the laser screens $Q_{1}$ and $Q_{2}$, as well as $T_{1}$ and $T_{2}$, are calculated respectively and shown in Table 2. The accuracy and consistency of measurement can be measured by uncertainty.

$\bar{v_{k}}$ is the *k*th armature at the velocity measuring point. $\bar{v_{k}}$ was defined as Formula (15) average velocity.

(15) $$\begin{array}{c} \bar{v_{k}}=\frac{v_{1k}+v_{2k}}{2} \end{array}$$

σ is uncertainty of $\bar{v_{k}}$, and is defined as Formula (16).

(16) $$\begin{array}{c} \sigma =\sqrt{\frac{{\left(v_{1k}-\bar{v_{k}}\right)}^{2}+{\left(v_{2k}-\bar{v_{k}}\right)}^{2}}{2}} \end{array}$$

A smaller value of σ indicates better consistency, higher reliability, and greater measurement accuracy of the velocity values between $v_{1k}$ and $v_{2k}$ that were measured by the two sets of velocity test system.

The data listed in Table 2 show the velocity $v_{1k}$ and $v_{2k}$ of the armature passing through the midpoint of two sets of laser velocity measurement systems according to the test principle shown in Figure 9. According to Formulae (15) and (16), the average velocity and uncertainty σ can be obtained.

The data in Table 2 are fitted. We use relative uncertainty to indicate the test system accuracy. $\sigma_{0}$ is the relative uncertainty of $\bar{v_{k}}$, $\sigma_{0}$ is defined as Formula (17).

(17) $$\begin{array}{c} \sigma_{0}=\frac{\sigma }{\bar{v_{k}}} \end{array}$$

The relative uncertainties $\sigma_{0}$ calculated from the uncertainty in Table 2 are less than 0.1%. It can be seen that the consistency of the velocity trend measured by the two test systems is very good, implying that the test data are highly credible and the algorithm is reliable.

## 6. Conclusions

In view of the problems such as the harsher test environment of the launch armature velocity of the electromagnetic gun, the complicated interferences to the armature signal may be caused by the factors such as vibration, flare, fragments, and particles. A few data processing approaches that combine EEMD with the CA have been studied and used to realize the adaptive denoising of the signal of armature passing through the laser screen, to identify the attribution of the corresponding signal of the armature, to obtain an effective signal of the armature, and to select the maximal slope point in the signal as a feature point to accurately extract the time information. As such, the flying velocity of the armature passing the screen can be calculated, which improves the accuracy of the test. The EEMD algorithm and CA proposed in this study are self-adaptive analysis methods, which are suitable for the calculation of the time interval for various shapes of objects that pass through two screens, and provide a high-precision approach to obtain the effective information about the armature passing through the laser screen, especially when no ideal waveforms can be detected under the circumstance where the system hardware is disturbed.

## Figures and Tables

**Table 1:** Velocity of armature passing laser screen.

Signals	1st	2nd	3rd	4th	5th	6th
Velocity (m/s)	991	970	932	888	974	682

**Table 2:** Calculation results of the velocity of armature passing through four laser screens.

Signals	1st	2nd	3rd	4th	5th	6th
$v_{1k}$ (m/s)	893.06	888.19	818.92	1259.04	1256.00	1031.64
$v_{2k}$ (m/s)	894.16	889.75	819.05	1260.85	1257.23	1031.67
$\bar{v_{k}}$ (m/s)	893.61	888.97	818.99	1259.95	1256.62	1031.66
Uncertainty (σ)	0.55	0.78	0.07	0.91	0.62	0.02
Relative uncertainty ($\sigma_{0}$)	0.062%	0.088%	0.008%	0.072%	0.049%	0.002%

## Data Availability

Not applicable.

## Abbreviations

The following abbreviations are used in this manuscript:

EEDM	Ensemble Empirical Mode Decomposition
CA	Correlation Algorithm
EMD	Empirical Mode Decomposition
IMF	Intrinsic Mode Function

## References

[1] Xu W.D., Yuan W.Q., Chen Y., Dai Y.B., Zhou Y., Yang D., Yan P., Li J. (2012). Sliding electrical contact performance of electromagnetic launcher system in rapid fire mode. Qiangjiguang Yu Lizishu.

[2] Zhao H., Xu W.D., Ma T.H., Yuan W.Q., Ding W.J. (2014). Arc Interference Suppressing Laser Screen Velocity Measurement of Electromagnetic Gun. Fire Control Command Control.

[3] Zhao D.G., Zhou H.C., Liu J., Zhang B., Luo Q.Q. (2013). High-precision velocity measuring system for projectiles based on retroreflective laser screen. Optik.

[4] Vargas R.N., Veiga A.C. (2021). Empirical Mode Decomposition, Viterbi and Wavelets Applied to Electrocardiogram Noise Removal. Circ. Syst. Signal Process..

[5] Wang H., Chen J., Dong G. (2014). Feature extraction of rolling bearing’s early weak fault based on EEMD and tunable Q-factor wavelet transform. Mech. Syst. Signal Process..

[6] Wang Y.H., Cheng S.H. (2022). Boundary Effects for EMD-Based Algorithms. IEEE Signal Process. Lett..

[7] Xu D., Shen G.Q., Qian Z.P. (2011). Research on separation for mixed signals based on ensemble empirical mode decomposition. J. Mil. Commun. Technol..

[8] Hao H.Y. (2013). Multi component LFM signal detection and parameter estimation based on EEMD-FRFT. Optik.

[9] Peng K., Guo H.Y., Shang X.Y. (2021). EEMD and Multiscale PCA-Based Signal Denoising Method and Its Application to Seismic P-Phase Arrival Picking. Sensors.

[10] Wu Z.H., Huang N.E. (2009). Ensemble empirical mode decomposition: A noise-assisted data analysis method. Adv. Adapt. Data Anal..

[11] Lang X., Liu Y., Zhang Y.F., Xie L., Horch A., Su H.Y. (2020). Denoising of Industrial Oscillation Data Using EEMD with CCA. IFAC-PapersOnLine.

[12] Hao H.Y., Li X.F., Liu M.J., Zhang F. (2012). Time-frequency Feature Extraction Method Based on EEMD and Cohen Class to Suppress Cross Terms. Yingyong Jichu Yu Gongcheng Kexue Xuebao.

[13] Lee D.H., Ahn J.H., Koh B.H. (2017). Fault Detection of Bearing Systems through EEMD and Optimization Algorithm. Sensors.

[14] Liu S.H., Hsieh C.H., Chen W.X., Tan T.H. (2019). ECG Noise Cancellation Based on Grey Spectral Noise Estimation. Sensors.

[15] Huang N.E., Shen Z., Long S.R., Wu M.C., Shih H.H., Zhang Q., Yen N.C., Tung C.C., Liu H.H. (1998). The empirical mode decomposition and the Hilbert spectrum for nonlinear and non-stationary time series analysis. Proc. R. Soc. Lond..

[16] Nie D.H., Xie K., Zhou F., Qiao G. (2020). A Correlation Detection Method of Low SNR Based on Multi-Channelization. IEEE Signal Process. Lett..

[17] He X., Cao Q.S. (2011). Development and Critical Techniques of Electromagnetic Launch Technology. J. China Acad. Electron. Inf. Technol..

[18] Xu W.D., Chen Y., Yuan W.Q., Zhao Y., Wang X.B., Yan P. (2014). Design of armature with high muzzle velocity in the small caliber electromagnetic launcher. Qiangjiguang Yu Lizishu.

